# Identification and validation of biomarkers based on cellular senescence in mild cognitive impairment

**DOI:** 10.3389/fnagi.2023.1139789

**Published:** 2023-04-28

**Authors:** Songmei Ma, Tong Xia, Xinyi Wang, Haiyun Wang

**Affiliations:** ^1^Department of Anesthesiology, The Third Central Clinical College of Tianjin Medical University, Tianjin, China; ^2^Department of Anesthesiology, The First People’s Hospital of Shangqiu, Shangqiu, Henan, China; ^3^Tianjin Key Laboratory of Extracorporeal Life Support for Critical Diseases, Tianjin, China; ^4^Artificial Cell Engineering Technology Research Center, Tianjin, China; ^5^Tianjin Institute of Hepatobiliary Disease, Tianjin, China

**Keywords:** mild cognitive impairment, cellular senescence, diagnostic model, biomarker, elderly patients

## Abstract

**Background:**

Mild cognitive impairment (MCI), a syndrome defined as decline of cognitive function greater than expected for an individual’s age and education level, occurs in up to 22.7% of elderly patients in United States, causing the heavy psychological and economic burdens to families and society. Cellular senescence (CS) is a stress response that accompanies permanent cell-cycle arrest, which has been reported to be a fundamental pathological mechanism of many age-related diseases. This study aims to explore biomarkers and potential therapeutic targets in MCI based on CS.

**Methods:**

The mRNA expression profiles of peripheral blood samples from patients in MCI and non-MCI group were download from gene expression omnibus (GEO) database (GSE63060 for training and GSE18309 for external validation), CS-related genes were obtained from CellAge database. Weighted gene co-expression network analysis (WGCNA) was conducted to discover the key relationships behind the co-expression modules. The differentially expressed CS-related genes would be obtained through overlapping among the above datasets. Then, pathway and GO enrichment analyses were performed to further elucidate the mechanism of MCI. The protein–protein interaction network was used to extract hub genes and the logistic regression was performed to distinguish the MCI patients from controls. The hub gene-drug network, hub gene-miRNA network as well as transcription factor-gene regulatory network were used to analyze potential therapeutic targets for MCI.

**Results:**

Eight CS-related genes were identified as key gene signatures in MCI group, which were mainly enriched in the regulation of response to DNA damage stimulus, Sin3 complex and transcription corepressor activity. The receiver operating characteristic curves of logistic regression diagnostic model were constructed and presented great diagnostic value in both training and validation set.

**Conclusion:**

Eight CS-related hub genes – SMARCA4, GAPDH, SMARCB1, RUNX1, SRC, TRIM28, TXN, and PRPF19 – serve as candidate biomarkers for MCI and display the excellent diagnostic value. Furthermore, we also provide a theoretical basis for targeted therapy against MCI through the above hub genes.

## 1. Introduction

Mild cognitive impairment (MCI), an intermediate stage between normal cognitive aging and dementia, is defined as poor cognitive performance on neurocognitive tests without significant impairment of instrumental activities of daily living (ADL). Etiologies associated with MCI mainly include degenerative and vascular processes, psychiatric causes, and some comorbidities like hypertension, diabetes mellitus and hyperlipidemia. The latest US Census data show that the adjusted prevalence of all-cause MCI was 22.7% (Rajan et al., 2021). With the follow-up duration lasting over 5 years, the annual rate of progression to dementia has been estimated to 5–15% (Dunne et al., 2021). So far, there is a roadblock to the early diagnosis and effective treatments due to the lack of consistency with screening for MCI and unclarified molecular mechanism. With the increasing aging population, the prevalence of MCI among the elderly warrants attention to explore the mechanism and identify the markers linked to the diagnosis of MCI. Such markers may offer researchers a point of intervention to minimize the future burden of dementia.

Cellular senescence (CS) is a stress response that can be induced by DNA injury, telomere dysfunction, organelle stress, among others, which is characterized by an essentially irreversible proliferative arrest, abnormal metabolism of mitochondria as well as lysosomes. Senescent cells have been recognized as a fundamental driving factor of some senile chronic diseases, cancer and other age-related diseases (Saez-Atienzar and Masliah, 2020; Wissler Gerdes et al., 2020; Cortesi et al., 2021). Senescent cells display three major characteristics – loss of proliferation or regeneration capacity, alteration of metabolic functions and resistance to apoptosis, and secretion of an array of pathogenically active molecules, termed senescence-associated secretary phenotype (SASP) (Khosla et al., 2020), a suite of pro-inflammatory cytokines which could not only improve tissue homeostasis through promoting myofibroblast differentiation as well as wound healing in young people (Baker et al., 2016), they generate a proinflammatory environment and recruit immune cells to accelerate degenerative changes (Muñoz-Espín and Serrano, 2014). When a lot of immune cells were brought to the brain, it would further induce neuroinflammation and exert an impact to cognitive performance (Gaikwad et al., 2021; Guerrero et al., 2021; Sikora et al., 2021). Besides, mitochondrial dysfunction can be often found in senescent cells, such changes would make energy metabolism in these senescent cells prone to be disordered and consequently generate more free radicals damaging senescent cells themselves or the cells around (Balaban et al., 2005). It is reported that age-related accumulation of senescent cells may dysregulate neural stem cells directly causing the decline of brain function in hippocampus, which may be a potential mechanism in MCI (Fatt et al., 2022). Moreover, the removal of these cells could significantly reduce the infiltration of peripheral immune cells into the brain parenchyma and alleviate aging-related inflammatory response and cognitive function (Zhang et al., 2022).

Neurodegenerative diseases such as Alzheimer’s disease (AD), Parkinson’s disease, and other types of dementia are more likely to occur as people age. Cell senescence was first discovered *in vitro* after large numbers of cultured fibroblasts were examined (Hayflick, 1965), and there is increasing evidence suggesting that cellular senescence plays an important role in age-related pathologies (Campisi and Robert, 2014; Muñoz-Espín and Serrano, 2014). Given the consideration of the important role cellular senescence plays in cognitive decline, we downloaded and analyzed the gene expression dataset about MCI (GSE63060) from GEO and senescence-related genes from the CellAge Database, hub genes associated with cellular senescence on MCI were identified in this study, 14 pairs of clinical samples were collected, patients with or without MCI were matched on the basis of age and education. Then multi-dimensional interaction networks were conducted to help researchers further understand the mechanism of MCI and develop novel treatments to alleviate the symptoms of MCI or slow its progression.

## 2. Materials and methods

### 2.1. Data source

All the microarray data after normalization were analyzed by R software. Microarray expression data for MCI patients and controls were downloaded from the Gene Expression Omnibus (GEO) database. The sample source of both GSE63060 and GSE18309 is from human blood. The GSE63060 dataset that included 99 controls and 80 MCI samples was used as training set, while GSE18309 dataset which included 3 controls and 3 MCI samples was used as external validation sets. 279 CS-related genes were acquired from Cell Age database.^1^

### 2.2. Acquirement of differentially expressed genes

Differential expression genes (DEGs) were analyzed between the controls and MCI patients using the “limma” package in R software, the cutoff values were adj.P.Val < 0.05. The heatmap cluster and the volcano plot about DEGs were created using the “pheatmap” and “ggplots” packages *via* R software.

### 2.3. Weighted gene co-expression network analysis

A weighted co-expression network was constructed for the expression profile data of the GSE63060 dataset with the help of the WGCNA package of R software (Langfelder and Horvath, 2008). Firstly, samples were clustered to assess the presence of any outliers. Then, the automatic network construction function was used to get the co-expression network. An ideal soft threshold (β) was selected and verified with the help of the “pick Soft Threshold” function. The matrix data were then transformed into an adjacency matrix, followed by clustering, to identify modules based on the topological overlap. After completing the calculation of module eigengene (ME) and merging similar modules in the clustering tree according to ME, a hierarchical clustering dendrogram was drawn. Modules were combined with phenotypic data to measure the significance of genes as well as clinical information, and analyze the correlation between modules and clinical features. In this way we could find out which modules are most relevant to MCI. After overlapping DEGs, module genes and CS-related genes, we obtained the final DEGs associated with CS-related genes (hereinafter referred to as CS-DEGs).

### 2.4. Functional annotation and pathway enrichment analysis

Functional enrichment analysis of CS-DEGs was performed in three domains of Gene Ontology (GO) database using the R package “clusterProfiler” (The Gene Ontology Consortium, 2017), including biological process (BP), cellular component (CC), and molecular function (MF). The Kyoto Encyclopedia of Genes and Genomes (KEGG) database contains datasets of pathways involving biological functions, diseases, chemicals, and drugs (Kanehisa and Goto, 2000). The enrichment analysis was carried out by “cluster Profiler” package to determine the biological functions of the genes and relevant pathways. *p* < 0.05 was considered statistically significant. “Omic Circos” package was used to analyze and display the location of genes on chromosomes.

### 2.5. Protein–protein interaction network construction

To further explore the interaction among the obtained CS-DEGs, we used the Search Tool for the Retrieval of Interacting Genes (STRING)^2^ to construct a PPI network (Szklarczyk et al., 2015). Then, Cytoscape plugin-Analyze Network was used to screen the significant modules with the degree >3 in the PPI network, and hub genes from CS-DEGs were obtained. The “ggpubr” package was used to perform Spearman correlation analysis on these hub genes.

### 2.6. Construction and validation of the logistic regression diagnostic model

To effectively differentiate the MCI from controls, the logistic regression diagnostic model was constructed using the glm function in R language (Friedman et al., 2010). The gene expression was seen as continuous variable and sample type as binary classification variable. The stepwise regression method was used to screen the variables with *p* < 0.05, and the final adjusted model was established based on the screened variables. The diagnostic and predictive performance to MCI of the logistic regression model would be evaluated by receiver operating characteristic (ROC) analysis.

### 2.7. Correlation and functional similarity analysis of hub genes and gene set enrichment analyses analysis

The “ggpubr” package was used to perform Spearman correlation analysis on hub genes. Moreover, the functional similarity among proteins was evaluated using the geometric mean of semantic similarities in CCs and MFs through the GOSemSim package (Yu et al., 2010). Functional similarity, the geometric mean of their semantic similarities in GO-MF and GO-CC terms, is designed for measuring the strength of the relationship between each protein and its partners by considering function and location of proteins. Moreover, GSEA of hub genes were performed using the “GSEABase” packages, KEGG gene sets were used as a reference, with *p* < 0.05 being a statistically significant difference.

### 2.8. Construction of gene-drug interaction network and regulatory network of hub genes

In order to explore the potential therapeutic drugs for MCI, drugs targeting proteins encoded by key genes were identified through the DGIdb database.^3^ Then we used NetworkAnalyst database^4^ and starBase databases^5^ to predict the transcription factors (TFs) and miRNAs related to hub genes. The association between hub genes and their TFs or miRNAs were integrated into a regulatory network using Cytoscape software.

### 2.9. Sample collection

This study was approved by the Medical Ethics Committee of the Third Central Clinical College of Tianjin Medical University (approval number: IRB2022-011-02), which complied with the Declaration of Helsinki. All subjects signed informed consent. Patients with the age of more than 65 years and undergoing lumbar decompression and fusion were enrolled. Total of 14 MCI patients and 14 controls were enrolled in the study, and subjects undergoing MCI were matched with healthy controls according to age and gender. All subjects underwent a structured interview and a battery of neuropsychological assessments, such as MMSE, MoCA and Clinical Dementia Rating (CDR). MCI was identified as follows: MMSE < 27 (17–27 for illiterate individuals, 20–27 for participants with elementary school education, and 24–27 for those with middle school education and above; Mitchell, 2009), MoCA scores in the range of 15–24 (Memoria et al., 2013), and CDR equal to 0.5.

### 2.10. RNA extraction and quantitative real-time-PCR

The blood samples were collected before surgery. The peripheral blood mononuclear cells (PBMCs) were extracted using Lymphocyte Separation Medium (Human) (P8610, Solarbio, Beijing, China), washed with 1 × PBS (FZ1258, Solarbio, Beijing, China) 3 times and then total RNA from PBMCs was extracted by the TRIzol reagent (15596026, Thermo Fisher Scientific Inc., Waltham, MA, United States). The Nano Drop 2000 Spectrophotometer (Thermo Fisher Scientific, Waltham, MA, United States) was utilized to measure the concentration and purity of the extracted RNA, with the A260/A280 between 1.8 and 2.0. The cDNA synthesis was conducted using the reverse transcription kit (RR036A, Takara, Japan). Using β-actin as a reference, we performed QRT-PCR with the SYBR Premix Ex Taq II (RR820A, Takara, Japan) on an ABI 7500 instrument (Applied Biosystems, Foster City, CA, United States), with 3 duplicates each well. Primer sequences (Sangon Biotech, Shanghai, China) for reference and candidate genes are shown in Table 1. The 2^−∆∆Ct^ method was applied to calculate the relative expression level of mRNA.

**Table 1:** Primer sequences for QRT-PCR.

Gene		Primer sequence (5′ → 3′)
SMARCA4	Forward	CAAAGACAAGCACATCCTCGCC
	Reverse	GCCACATAGTGCGTGTTGAGCA
SMARCB1	Forward	GGCATCAGAAGACCTACGCCTT
	Reverse	CTCCATCTCAGCGTCTGTCAGA
RUNX1	Forward	CCACCTACCACAGAGCCATCAA
	Reverse	TTCACTGAGCCGCTCGGAAAAG
SRC	Forward	CTGCTTTGGCGAGGTGTGGATG
	Reverse	CCACAGCATACAACTGCACCAG
TRIM28	Forward	CAAGATTGTGGCAGAGCGTCCT
	Reverse	CATAGCCTTCCTGCACCTCCAT
TXN	Forward	GTAGTTGACTTCTCAGCCACGTG
	Reverse	CTGACAGTCATCCACATCTACTTC
PRPF19	Forward	TGGGCTTTCTCTGACATCCAGAC
	Reverse	CCTGTTCCAAAGATGAGTCCGTC
β-actin	Forward	CACCATTGGCAATGAGCGGTTC
	Reverse	AGGTCTTTGCGGATGTCCACGT

## 3. Results

### 3.1. Identification of DEGs and co-expression network construction

**Figure 1:**
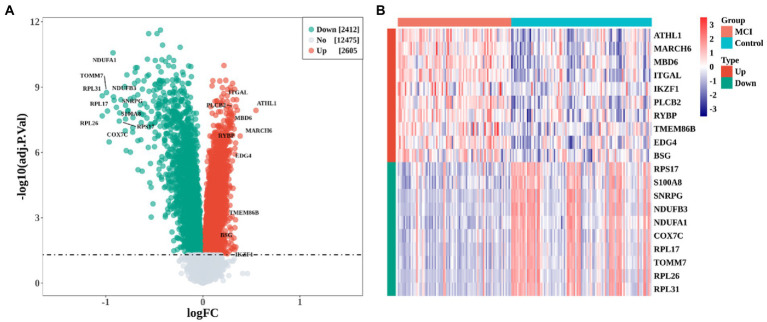
Identification of differentially expressed genes (DEGs) between MCI and Control group. **(A)** Volcano plot of DEGs expression profile. **(B)** heatmap of the top 10 up-regulated and down-regulated DEGs, respectively.

A total of 5,017 DEGs, including 2,605 up-regulated genes and 2,412 down-regulated genes, were obtained when comparing MCI cases to controls (Figure 1A, adj.P.val < 0.05). The heatmap showed the most significant 10 up-regulated and down-regulated genes (Figure 1B).

**Figure 2:**
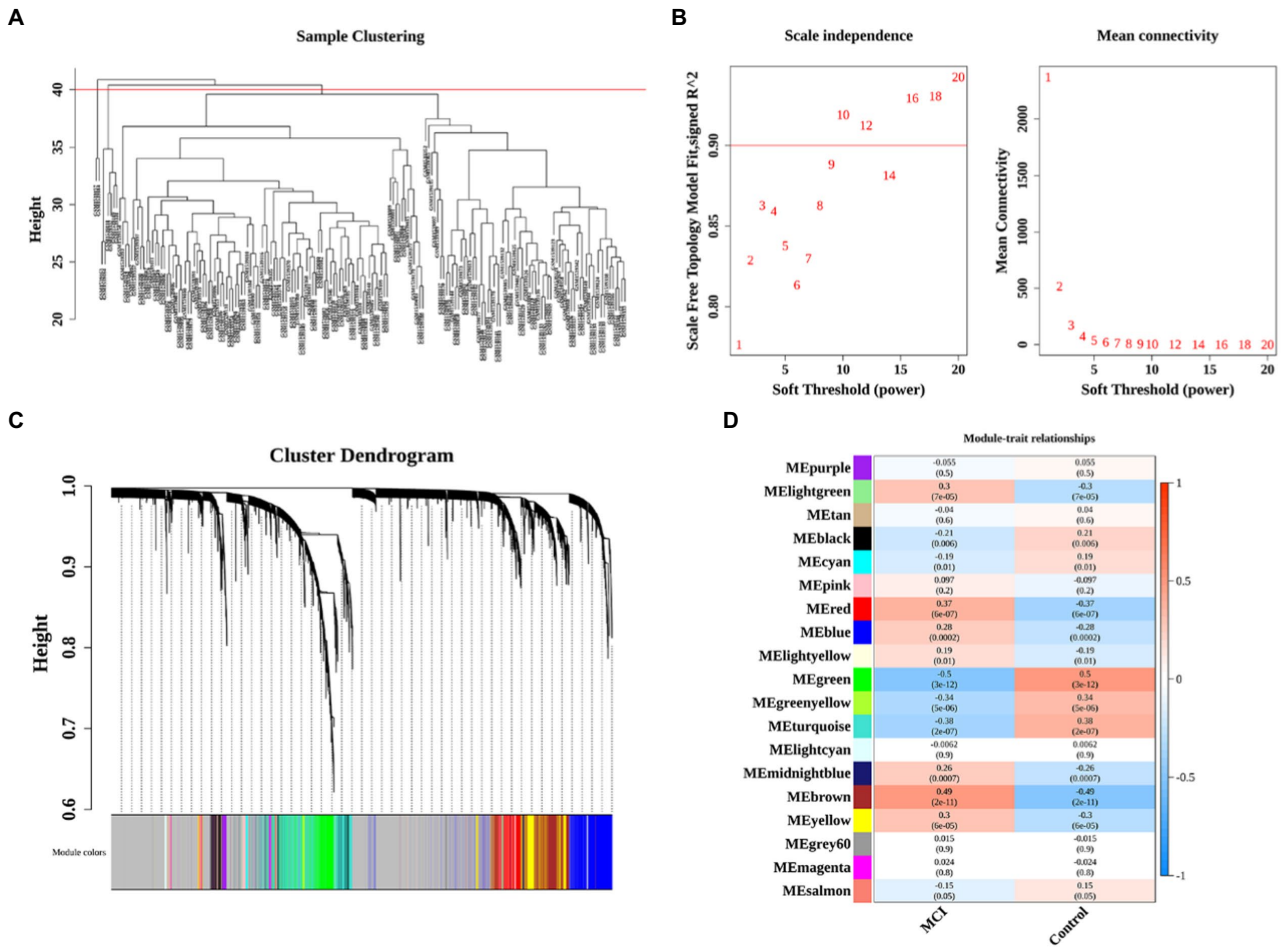
Identification of significant modules and genes of GSE63060 by WGCNA. **(A)** Clustering dendrogram of samples. **(B)** Network topology analysis with different soft thresholds. The scale-free *R*^2^ was 0.90 and the soft threshold was 10. **(C)** A cluster dendrogram of module-specific colors showed 19 co-expressed gene modules between MCI and controls. **(D)** Heatmap of correlation between disease groupings and gene modules.

The co-expression network was constructed by WGCNA to further identify genes strongly associated with MCI, followed by clustering of samples and 8 outliers need to be culled (Figure 2A). A soft threshold of β = 10 was selected for consistency with the scale-free network. 19 modules were identified based on average hierarchical clustering and dynamic tree clipping (Figures 2B,C). The correlations of the above 19 modules with MCI and controls were presented *via* heat maps, with two key modules including green and brown contained a total of 2,394 genes demonstrating the highest correlation with MCI (cor = −0.5, *p* < 0.001 in green module; cor = 0.49, *p* < 0.001 in brown module; Figure 2D).

### 3.2. Go and KEGG enrichment analysis of CS-DEGs

**Figure 3:**
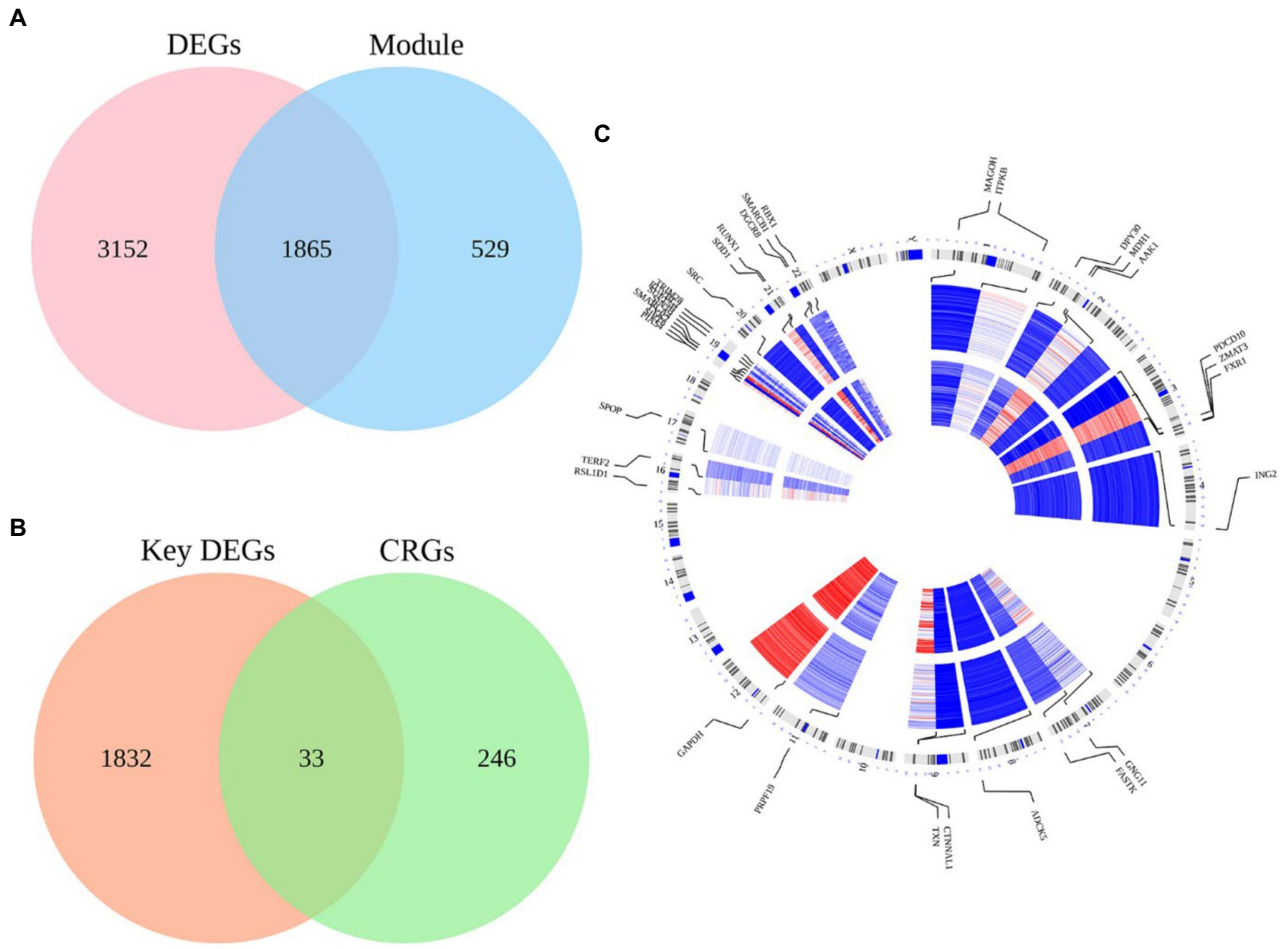
The intersection of MCI-related modules and cellular senescence-related genes as well as their locations on chromosomes. **(A)** There were 5,017 DEGs in the GSE63060 dataset and 2,394 genes in the blue module of the GSE63060 dataset, and 1865 genes were obtained as key DEGs by overlapping the two datasets. **(B)** There were 1865 key DEGs and 279 cellular senescence-related genes (CRGs) from Cell Age database, and 33 genes were obtained as CS-DEGs by overlapping the two datasets. **(C)** Chromosome locations of the 33 CS-DEGs.

We overlapped 2,394 module genes and DEGs to obtain 1865 MCI-related DEGs (Figure 3A). Furthermore, after intersecting the CS-related genes (CGRs) with MCI-related DEGs, we found total 33 CS-DEGs (Figure 3B). Localization analysis revealed that these 33 CS-DEGs were mainly located in 14 pairs of chromosomes (Figure 3C).

**Figure 4:**
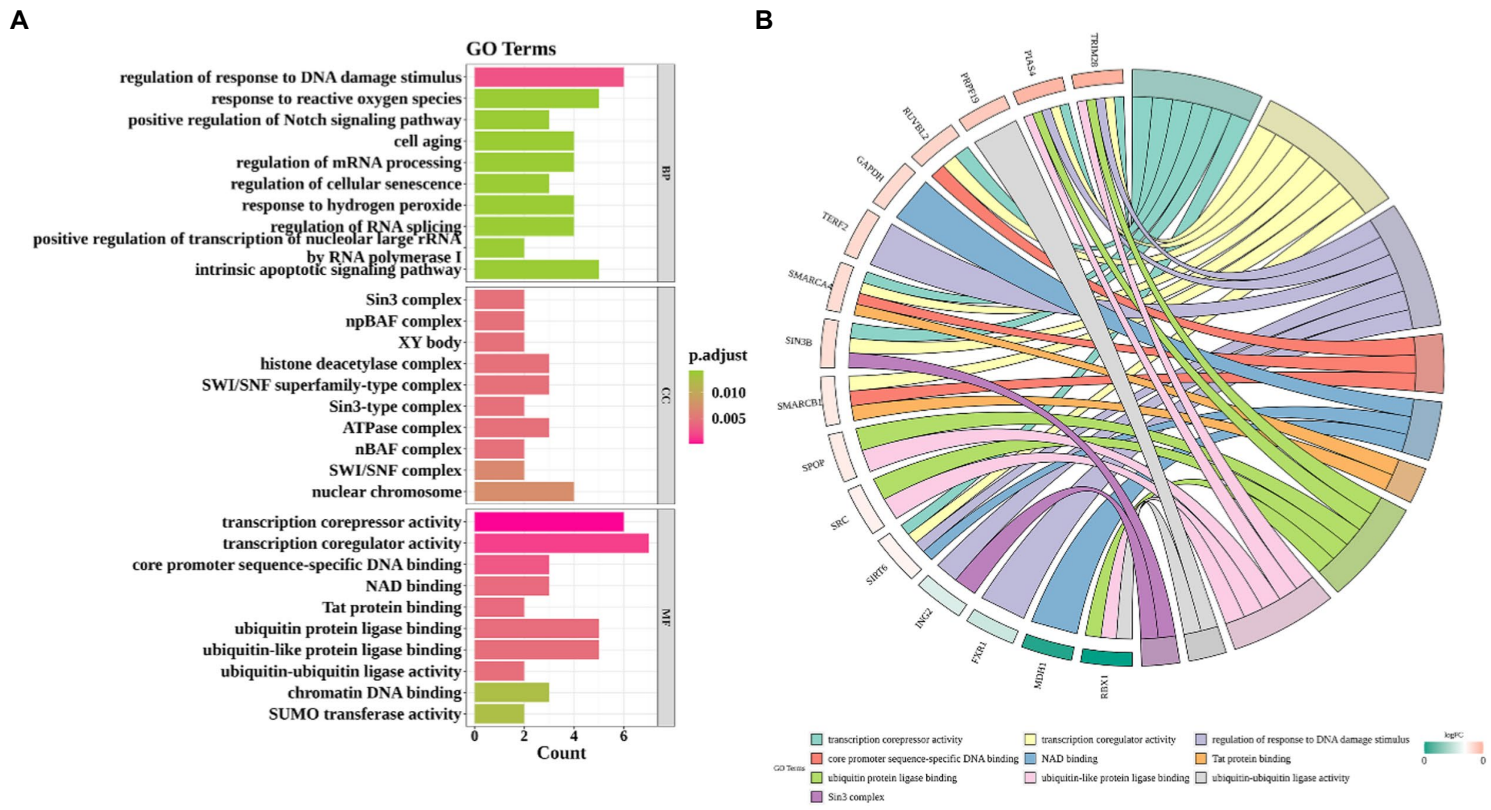
Gene ontology enrichment analysis. **(A)** Enrichment bar graph. X-axis indicates the number of CS-DEGs. Y-axis represents GO terms. All GO terms are grouped into three ontologies: biological process (BP), cellular component (CC) and molecular function (MF). The color of bars represents the adj.P.Val. **(B)** Chord plot shows that the links were straight forward to show the relations between genes and some key GO terms.

In GO analysis, the above 33 CS-DEGs were classified into biological process (BP), cellular component (CC), and molecular function (MF). Among BP classes, CS-DEGs were mainly enriched in the response to DNA damage stimulus and reactive oxygen species. In the CC category, the genes expressed were mostly enriched in transcription corepressor activity, transcription coregulator activity and core promoter sequence-specific DNA binding. The pathways enriched by GO-MF were principally associated with Sin3 complex, npBAF complex and XY body (Figure 4A). GO enrichment analysis showed that CS-DEGs were mainly enriched in transcription corepressor activity, transcription coregulator activity and response to DNA damage stimulus (Figure 4B). Next, we performed KEGG pathway enrichment analysis to further understand gene biological functions of CS-DEGs and it did not reveal significantly enriched pathways.

### 3.3. PPI network construction and module analysis

**Figure 5:**
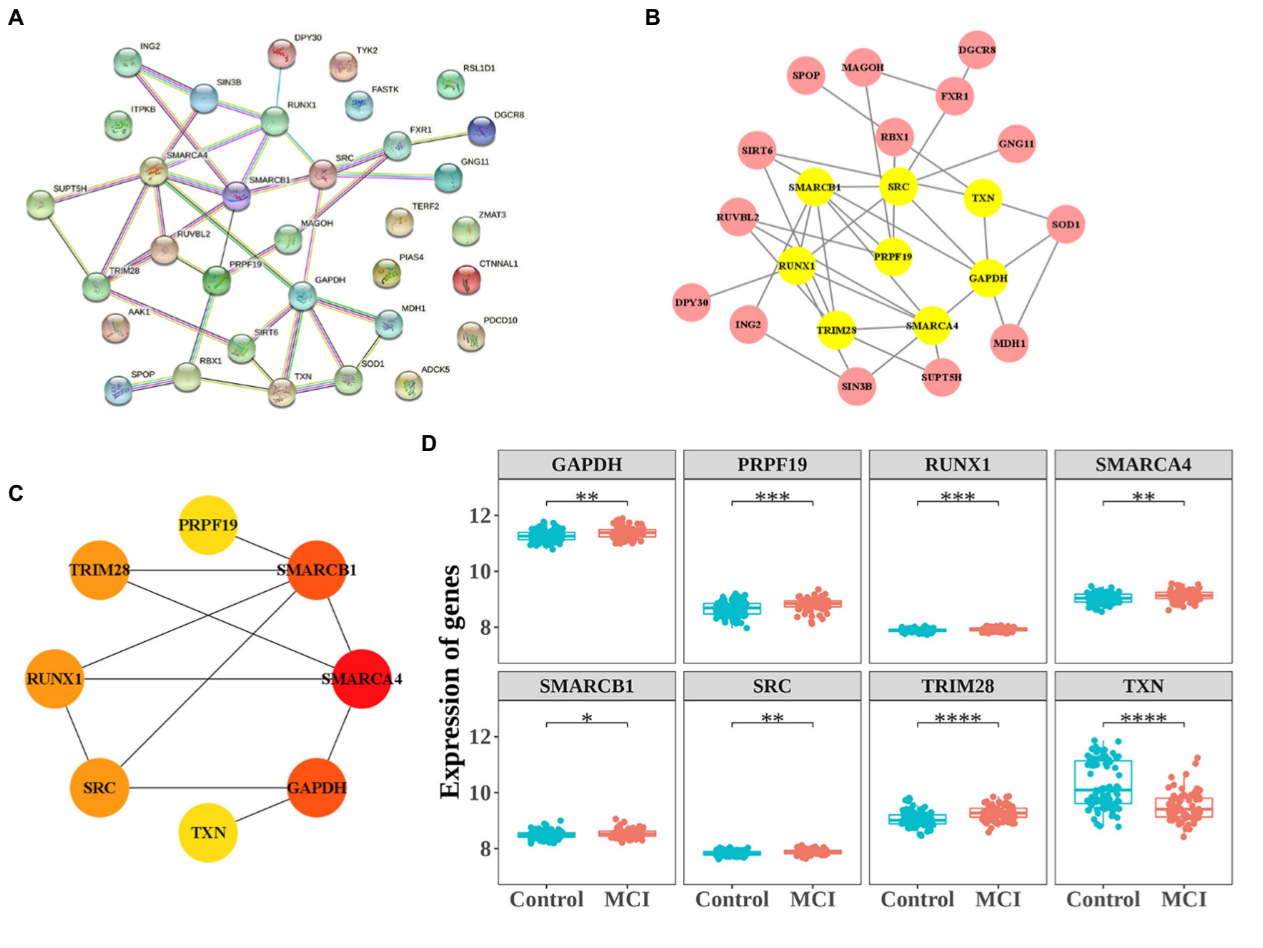
Protein–protein interaction (PPI) network construction and the expression of 8 hub-genes. **(A)** PPI network constructed with the CS-DEGs. **(B,C)** The significant module identified from the PPI network using the molecular complex detection (MCODE) method. **(D)** The expressions of 8 hub-genes. *p < 0.05, **p < 0.01, ***p < 0.005.

To further analyze the interaction of 33 CS-DEGs, Analysis of PPI network was performed using STRING and visualized by Cytoscape under default parameters (Figure 5A). By using Analyze Network plugin, the 8 genes – SMARCA4, GAPDH, SMARCB1, RUNX1, SRC, TRIM28, TXN, and PRPF19 – were selected as potential hub genes (Figures 5B,C). The expressions of all genes except TXN were upregulated in MCI and all displayed difference between groups (Figure 5D).

### 3.4. The logistic regression model for the diagnosis of MCI

**Figure 6:**
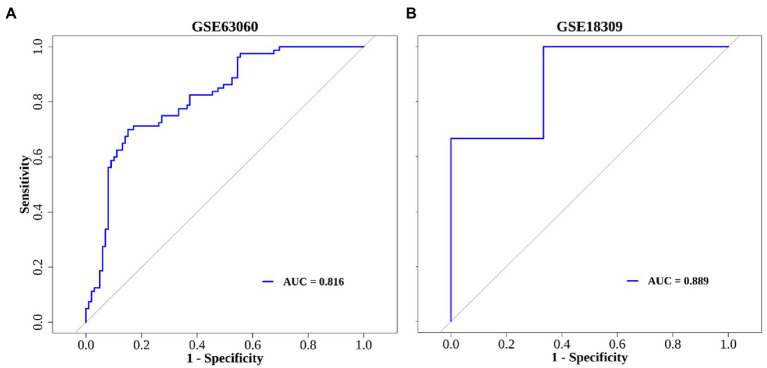
The ROC curve of diagnostic model. **(A)** GSE63060 dataset serves as the training set. **(B)** GSE18309 dataset serves as the validation set.

In order to further elucidate the clinical predictive value of the 8 hub genes, we applied the logistic regression model. Taken together, the logistic regression model established based on the hub genes could effectively identify individuals with or without MCI. The results of ROC curve in training set (AUC = 0.816) and validation set (AUC = 0.889) show the great predictive power of the model and suggest that these eight genes may be potential treatment targets for patients with MCI (Figures 6A,B).

### 3.5. Analysis of interaction effect and functional similarity for hub genes

**Figure 7:**
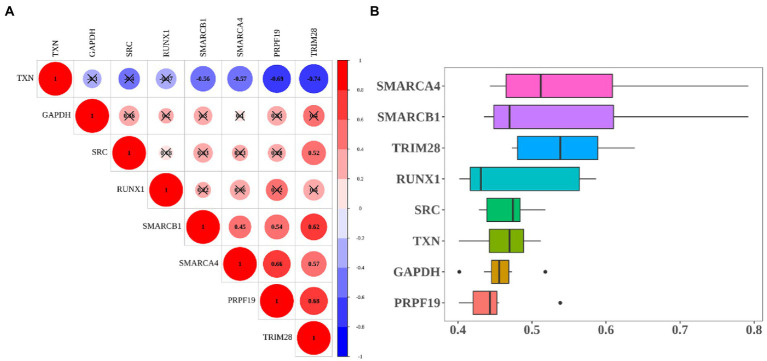
Heatmap of correlation and functional similarity among hub-genes. **(A)** Correlation matrix for all 8 hub genes. Some hub genes were negatively related, represented in blue, and others were positively related, represented in red. The darker the color, the higher the correlation was (*p* < 0.05). **(B)** Functional similarity of all 8 hub genes.

The correlation results showed that TXN had the negative correlation with other hub genes. Among them, TXN had the strong negative correlation with TRIM28 (cor = −0.74), the next was PRPF19 (cor = −0.69). PRPF19 and TRIM28 had the strongest positive correlation (cor = 0.68), followed by the correlation between SMARCB1 and PRPF19 (cor = 0.62; Figure 7A). Moreover, we ranked hub genes based on the average functional similarity relationships among proteins within the interactome of 8 hub genes. SMARCA4, SMARCB1 and TRIM28 were the three top-ranked proteins potentially playing key roles in MCI (Figure 7B).

**Figure 8:**
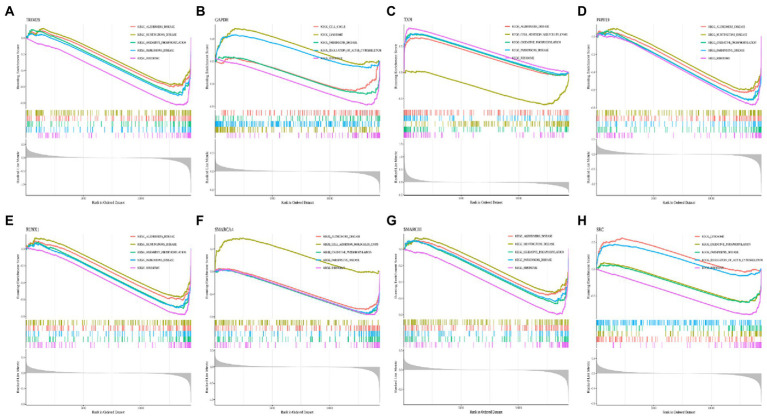
Enrichment analysis of pathway and gene ontology (GO) involved hub genes. **(A–H)** Gene Set Enrichment Analysis (GSEA) of TRIM28, GAPDH, TXN, PRPF19, RUNX1, SMARCA4, SMARCB1 and SRC. Different colors represent different signaling pathways.

By performing GSEA analysis of each hub gene, we found that all hub genes were associated with “KEGG_RIBOSOME” and “KEGG_PARKINSONS_DISEASE.” The top five important pathways associated with PRPF19 and RUNX1 were the same. PRPF19, RUNX1, SMARCA4, SMARCB1, SRC, TRIM28, and TXN were related to “KEGG_OXIDATIVE PHOSPHORYLATION.” PRPF19, RUNX1, SMARCA4, SMARCB1, TRIM28, and TXN were associated with “KEGG_ALZHEIMERS_DISEASE” (Figures 8A–H). These indicated that MCI shares common features with AD and Parkinson’s disease.

### 3.6. Construction of hub gene-drug interaction network and regulatory network of hub genes

**Figure 9:**
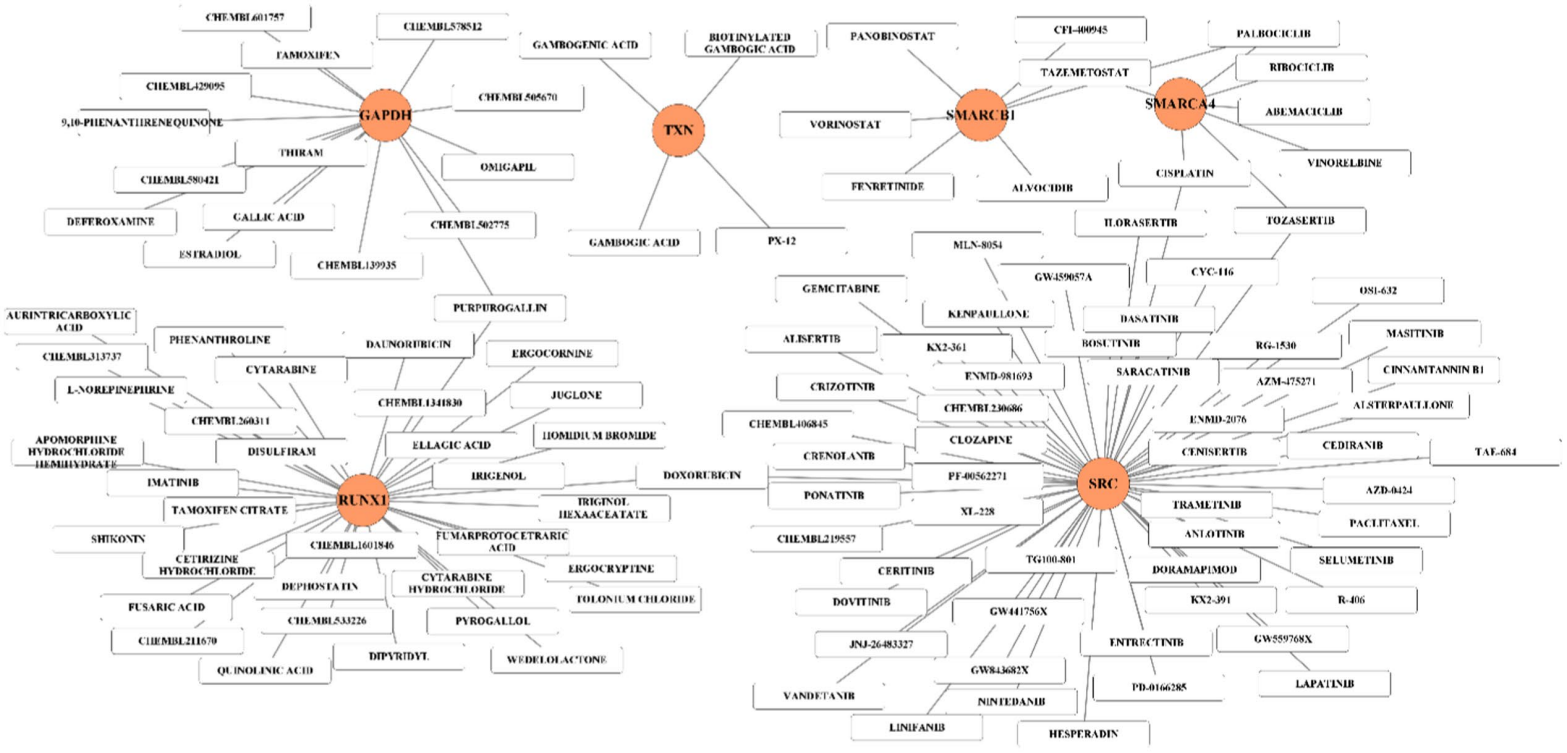
Hub gene-drug interaction network. The orange circles represent hub-gene and white rectangles represent small molecule drugs.

**Figure 10:**
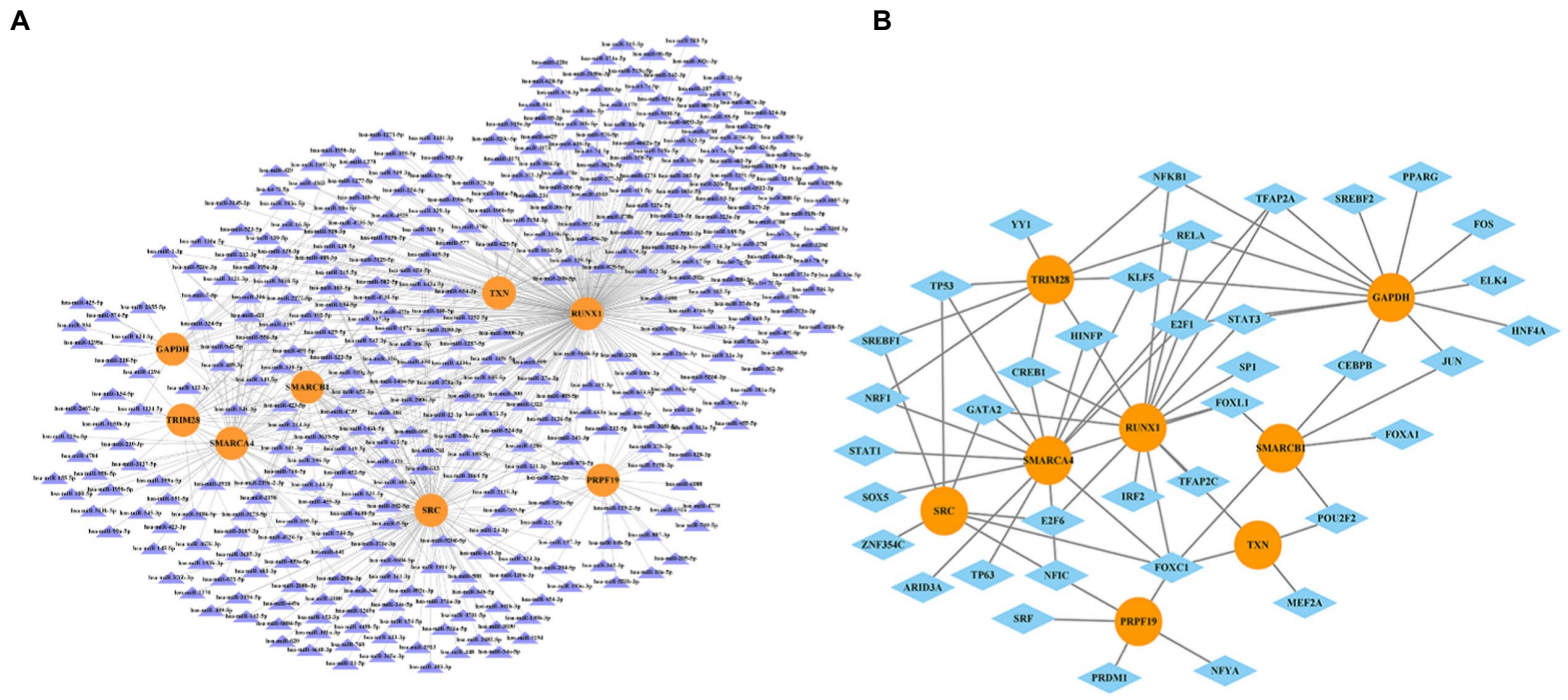
The miRNA-gene network **(A)** and transcription factor-gene regulatory network **(B)**. The orange circles represent hub-genes, purple triangles represent miRNAs and blue diamonds represent TFs.

In order to explore the interaction between 8 hub genes and potential treatment of MCI, drugs associated with the key genes were identified by DGIdb database. The interested genes (SMARCA4, GAPDH, SMARCB1, RUNX1, SRC, TRIM28, TXN, and PRPF19) are used as search terms and determined the extant drugs targeting these genes. Plenty of drugs could affect the expression of these eight hub genes except for PRPF19 and TRIM28, and a total of 124 drug-gene interaction pairs are in this network. Among them, SRC, RUNX1 and GAPDH were the top 3 hub genes targeted by 56, 35, 15 drugs, respectively (Figure 9).

we entered the 8 hub genes (SMARCA4, GAPDH, SMARCB1, RUNX1, SRC, TRIM28, TXN, and PRPF19) in starBase and NetworkAnalys database as search terms, and the searched results about miRNAs-genes and TFs-genes network were constructed by Cytoscape software. Among them, miRNAs targeting at least three hub genes were selected to construct the network. Finally, the network composed of 8 hub genes, 385 miRNAs and 39 TFs were established (Figure 10).

**Figure 11:**
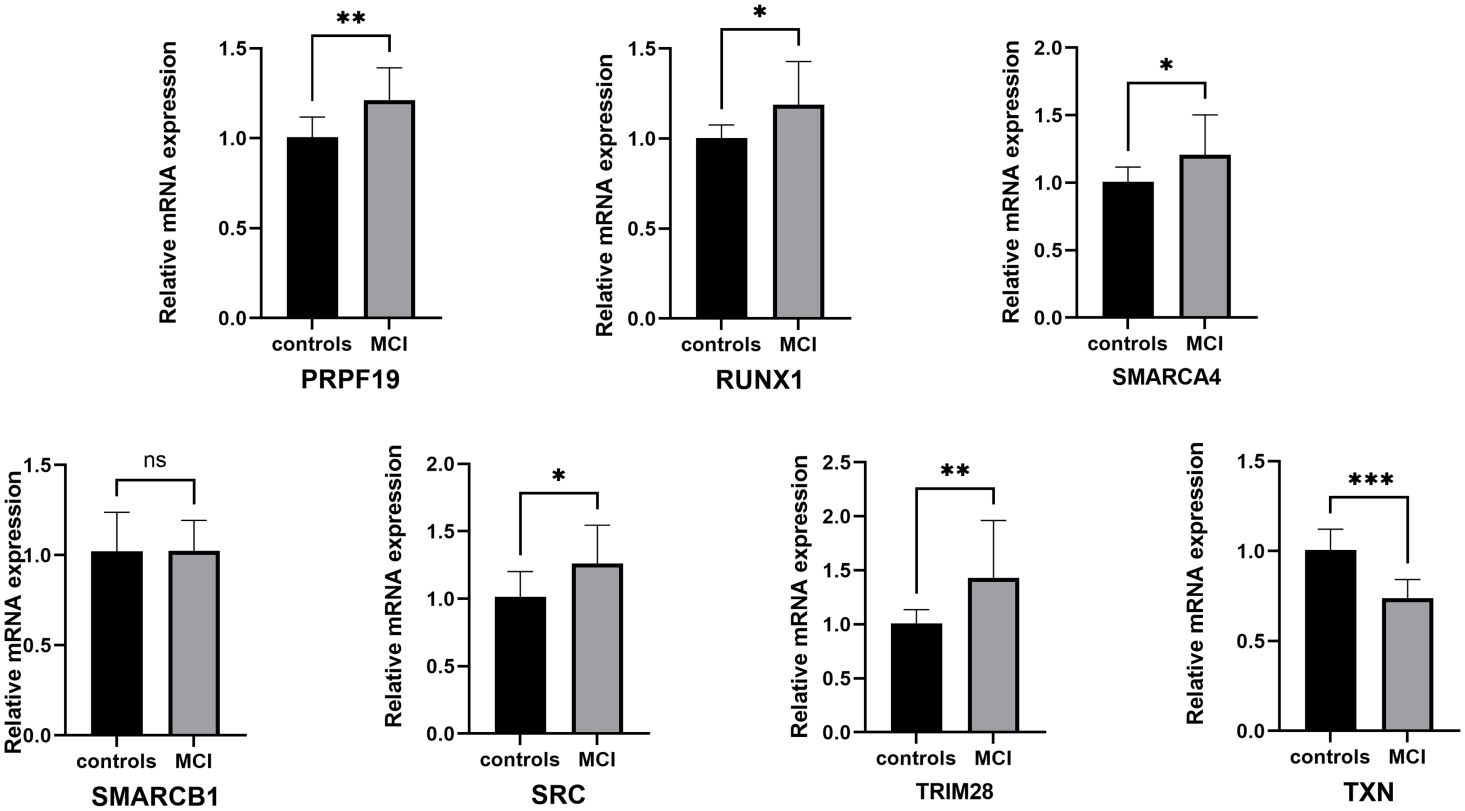
QRT-PCR validation of the hub genes between controls and MCI. The data were expressed as mean ± SEM (short for standard error of mean). *p < 0.05, **p < 0.01, ***p < 0.001, ****p < 0.0001, ns means no significance.

Then we recruited patients for clinical study to validate the above findings. After analyzing the basic demographic data for the enrolled patients, we found there were no differences in age, gender, BMI, ASA and preoperative comorbidity among patients with or without MCI, while educational attainment and neuropsychological test shows statistic difference, which was consistent with our diagnostic criteria for MCI (Table 2). The transcriptional changes of hub genes were detected in the PBMCs from patients with or without MCI by QRT-PCR (Figure 11). The results indicated that the expression levels of TXN was decreased in MCI group in comparison with those in controls, while PRPF19, RUX1, SMARCA4, SRC and TRIM28 were increased in MCI group, which was basically in line with GSE63060 and GSE18309. However, the expression level of SMARCB1 shows no statistical difference between MCI group and non-MCI group.

**Table 2:** Demographic data for patients with/without MCI.

	non-MCI (*n*=14)	MCI (*n*=14)		t/Z/χ^2^	*P* value
**Preoperative data**
Male sex, *n* (%)	50.00% (*n*=7)	50.00% (*n*=7)		0.000	1.000
Age (yr)	69.50 (64.75, 73.50)	67.50 (66.00, 70.25)		−0.554	0.603
BMI (kg/m^2^)	25.95 (23.94, 27.12)	27.34 (24.28, 28.92)		−0.827	0.427
ASA (II/III), *n*	10/4	9/5		0.164	0.686
Education level (yr)	12.00 (9.00, 12.00)	7.00 (3.75, 9.00)		−2.802	0.005^*^
**Comorbidity, before operation**	
Hypertension, n (%)	57.14% (*n*=8)	64.29% (*n*=9)		0.150		0.699	
Diabetes, *n* (%)	14.29% (*n*=2)	28.57% (*n*=4)		0.848		0.357
Coronary Artery Heart Disease, *n* (%)	14.29% (*n*=2)	21.43% (*n*=3)		0.243		0.622
**Neuropsychological test**	
MMSE	29.00 (28.00, 29.00)	24.00 (22.75, 25.25)		−4.425	0.000^*^
MoCA	25.00 (22.75, 26.25)	20.00 (16.00, 22.00)		−4.020	0.000^*^

Descriptive results of continuous variables were presented as the means ± standard deviations, median with inter-quartile range or numbers with percentages. The *P*-value is calculated by the independent-samples *T* test, Kruskal-Wallis H test or chi-square test, respectively. ^*^p<0.05 compared with group non-MCI.

MCI, mild cognitive impairment; BMI, Body Mass Index; ASA, American Society of Anesthesiology; MMSE, mini-mental state examination; MoCA, Montreal Cognitive Assessment.

## 4. Conclusion

This work has shown that the mRNA levels of SMARCA4, GAPDH, SMARCB1, RUNX1, SRC, TRIM28, TXN and PRPF19 in PBMCs are effective diagnostic biomarkers in MCI. Multifactorial interaction networks related to CRGs have been successfully constructed and these results would largely contribute to the understanding of the pathogenesis of MCI and provide the foundation to explore novel therapeutic targets for MCI.

## 5. Discussion

The rate of mean annual conversion to dementia in MCI (10%) is far higher than that in the general population (1–2%) (Yan et al., 2022). With the consideration of reversible characteristic of MCI, this stage would provide an optimal window to the early prevention and intervention of dementia. Thus, potential markers for diagnosis and treatment with high efficiency are urgently demanded. In the present study, we identified hub genes related to cellular senescence and developed a diagnostic model for MCI. Gene expression microarray data from GSE18309 and clinical samples were used to further verify the value of this model. We found that 8 biomarkers – SMARCA4, GAPDH, SMARCB1, RUNX1, SRC, TRIM28, TXN and PRPF19 – could identify individuals with MCI with ROC curve in training set (AUC = 0.816) and validation set (AUC = 0.889).

In this study, people with or without MCI were compared to identify differentially expressed genes, and 1,865 genes differentially expressed in key modules were identified based on WGCNA. We identified gene sets with similar expression patterns and analyzed the relationship between gene sets and sample phenotypes based on WGCNA analysis of key module genes, MCI was the sample phenotype compared to the control group, MCI correlated genes were identified as genes associated with the disease, the expression differences of individual genes are not considered in this analysis, thus, genes differentially expressed between MCI samples and controls were screened. Then the 1865 DEGs intersect with genes involved in cellular senescence, from the intersection of the DEGs with the genes involved in cellular senescence, DEGs associated with cellular senescence were identified. GO and KEGG enrichment analyses were performed to explore interactions among these 33 CS-DEGs which appeared to be associated with cellular senescence and MCI *via* DNA damage repair and histone deacetylation. According to the results of GO-BP, CS-DEGs were mainly enriched in regulation of DNA damage response, reactive oxygen species (ROS) response and positive regulation of oxygen species response. ROS are a group of short-lived, highly reactive, oxygen-containing molecules, which are necessary for the communication between nucleus and mitochondria but have detrimental effects at extremely high levels *via* damaging DNA, activating DNA damage response (DDR) (Valko et al., 2007). Many molecular processes can be affected by DNA damage and the cellular DDR, which would alter cell fate, deregulate intercellular communication, mutations or chromosomal aberrations caused by DNA damage, and then trigger genome instability as well as cellular senescence (Williams and Schumacher, 2016; Mladenov et al., 2018; Petr et al., 2020). In addition the damage of genes encoding for critical neuronal functions could affect synaptic plasticity, learning as well as memory formation (Kim et al., 2022; Miller et al., 2022; Yoon et al., 2022). Moreover, the high level of ROS as well as subsequent DNA damage would make the brain exposed to oxidative stress and such pathologic changes would increase the accumulation of amyloid-β as well as neurofibrillary tangles, neuronal loss and microglia activation, resulting in the occurrence and progression of neurodegenerative diseases, such as MCI, AD, among others (Akterin et al., 2006; Ghosh et al., 2015; Buccellato et al., 2021). In terms of the results of GO-MF and GO-CC, most of genes or pathways are principally associated with the regulation of cell cycle and the self-renewal or proliferative capacity of cells (Simone, 2006; Gupta et al., 2020; Braun et al., 2021; Mitra et al., 2022). In term of MF, mounting evidence showed that sequence-specific activators and repressors interact with coregulators, which in turn either stimulate or inhibit the binding or function (or both) of some transcription complex (Mannervik et al., 1999). As core component of transcriptional corepressor complex, Sin3 plays a transcriptional inhibitory role by binding to histone deacetylase (HDAC) (Kadamb et al., 2013). Sin3/HDAC complex have been implicated in learning and memory, whose dysregulation has been linked to cognitive impairment in brain aging and neurodegenerative diseases (Gräff et al., 2012). with the help of Sin3, HDAC would be promptly recruited to break sites to catalyze H3K56 as well as H4K16 deacetylation when confronting DNA double-strand breaks (DSBs) and stimulate DSB repair through the nonhomologous end joining (NHEJ) pathway. Therefore, it exerts an important regulatory influence on individual learning and memory through the changes of synaptic plasticity, dendritic spine density and synapse number (Fischer et al., 2007; Guan et al., 2009; Gräff et al., 2012; Cummings et al., 2018). The ATP-dependent chromatin remodeling complex BAF, namely SWI/SNF, mainly consists of SMARCB1, SMARCA4, SMARCA2, SMARCE1, ARID1A as well as ARID1B (Santen et al., 2013), and is crucial for the regulation of gene expression and differentiation. It is reported that npBAF is essential for neural development whose subunits have an enormous influence on dendritic morphogenesis, neuronal subtype maturation and learning as well as long-term memory (Alfert et al., 2019).

From the CS-DEGs, we identified genes with ≥10 connections in the PPI network analysis as hub genes. 8 hub genes were identified, which included SMARCA4, GAPDH, SMARCB1, RUNX1, SRC, TRIM28, TXN and PRPF19, among which all hub genes were up-regulated in MCI except for TXN. Logistic regression model was constructed to predict the risk of MCI (AUC = 0.816 in training set, 0.889 in validation set), which revealed a high diagnostic value for MCI. TXN is involved in many antioxidized and antiapoptotic reactions, at least two isoforms of which have been characterized in mammals, TXN1 (mainly in cytosolic) and TXN2 (mainly in mitochondrial). TXN contributes to the response to intracellular nitric oxide (NO) *via* the reversible S-nitrosylation of cysteine residues in CASP3, and thereby inhibits caspase-3 activity (Mitchell et al., 2007; Glaser et al., 2008). Besides, TXN-1 is required for nerve growth factor-mediated signal transduction and neurite outgrowth, and is involved in synaptic protein expression induced by BDNF (Bai et al., 2019). Thus, TXN is a promising early biomarker of AD, AD patients’ hippocampus tissue sections displayed abnormal TXN1 immunoreactivity and expression pattern, as promising early biomarkers of AD compared to controls (Arodin et al., 2014). TXN had strong negative correlation with TRIM28, which means TRIM28 would play a protective role in the pathogenesis of MCI. TRIM28 could bind to zinc finger proteins containing KRAB, acts as a transcriptional cofactor and an E3 ubiquitin ligase. The phosphorylation of TRIM28 at S473 promoted association between TRIM28 and cap binding complex dependent translation initiation factor (CTIF), which inhibits aggresome formation (Chang et al., 2021). It has been found that TRIM28 could regulate α-Syn and tau levels *via* SUMOylation and is highly expressed in neurodegenerative brain tissues (Rousseaux et al., 2016, 2018). As another Ubiquitin-protein ligase, pre-mRNA processing factor 19 (Prpf19/prp19) mainly involved pre-mRNA splicing and plays a neuroprotective role in the DDR. PRPF19 was upregulated during neurodegeneration, for it can antagonize exocyst complex component 7 (Exoc7) to improve neurodegeneration by degrading misfolded proteins in neurons through the ubiquitin-proteasome system, therefore, patients with MCI are often accompanied by higher PRPF19 levels than patients with normal cognitive function (Chen et al., 2021). SRC is a tyrosine-protein kinase whose activity can be inhibited by phosphorylation by c-SRC kinase. c-SRC and other tyrosine-phosphorylating protein kinases with a similar structure to c-SRC gave rise to the concept of Src family kinases (SFKs), SFK stimulation has been associated with microglial activation and neuropathological conditions, including Alzheimer’s and Parkinson’s. Fyn, an Src family non-receptor tyrosine kinase, has been linked to synaptic plasticity, which is a cellular mechanism for learning and memory. Fyn could upregulate amyloid-beta peptides (Aβ) production and mediate Aβ-induced synaptic deficits and neurotoxicity, in addition, Fyn could also induce tau tyrosine phosphorylation and then form neuroillary tangles (Bhaskar et al., 2005; Yang et al., 2011). The amyloid plaques and neurofibrillary tangles would finally lead to neurotoxicity and cognitive impairment (Haass and Mandelkow, 2010). RUNX1 belongs to master regulators for the age-dependent microglia module and increases the level of G9a through histone lysine methylation, leading to the increase of neuroinflammatory markers such as interleukin-6, tumor necrosis factor-α and then cognitive impairment in rats (Grinan-Ferre et al., 2019; Li et al., 2020). GAPDH is an enzyme known for its propensity to form aggregates when oxidized or complexed with mutant proteins and the amount of soluble or insoluble GAPDH complexes with Aβ in the CSF from patients with various stages of AD directly correlated with the severity of the disease (Gerszon and Rodacka, 2018; Lazarev et al., 2021). Thus, GAPDH is a promising pharmacological target for AD.

In order to further explore the possibility of these eight hub genes as potential therapeutic targets for MCI, we analyzed the interaction between the hub genes and available therapeutic drugs of MCI and found that numbers of drugs could affect the expression of these hub genes.

The DGIdb database was used to identify compounds targeting the proteins encoded by hub genes and 124 drugs were finally found. Natural organic acids may exert beneficial effects to improve cognition through increasing energy metabolism, taking part in antioxidant and anti-inflammatory, and thereby reducing damage and death of neural cells (Kolker et al., 2008; Colin-Gonzalez et al., 2015). Nutritional supplementation of gallic acid preserve the morphological and physiological integrity of the hippocampus against environmental neurotoxins by mopping up free radicals associated with oxidative stress induced AD (Ogunlade et al., 2022). Estradiol, has been widely reported owing to their neuroprotective action because they give rise to a wide range of cell signals and generate effects in genes by means of canonical pathways or through non-conventional mechanisms that are involved in neuronal survival, dendritogenesis and synapse remodeling (Barrera Ocampo et al., 2008). The Src family kinase inhibitor, saracatinib, targeting Fyn as a therapeutic intervention in AD, based on its activation by Aβ *via* cellular prion protein but also due to its known interaction with tau, uniquely linking the two key pathologies in AD. Bosutinib also increased survival *in vitro* of ALS iPSC-derived motor neurons from patients with sporadic ALS, bosutinib treatment modestly extended survival of a mouse model of ALS with an SOD1 mutation, suggesting that Src/c-Abl may be a potentially useful target for developing new drugs to treat ALS (Imamura et al., 2017). Drugs known to have effects on other neurodegenerative diseases could provide a new direction for MCI research.

Micro-RNAs (miRNAs) play a critical role in regulating gene expression. They would bind to specific messenger RNAs (mRNAs) and inhibit their translation as well as related protein synthesis, which is important for many cellular processes, including development, differentiation, and apoptosis. For instance, hsa-miR-212-5p could influence cognition by inhibiting SRC in our study, it is because hsa-miR-212-5p reduces neuronal synaptic plasticity by downregulating BDNF, MECP2, CREB and PTEN. The Inhibition of hsa-miR-212-5p would increase the expression of these proteins and improve cognitive performance, such as learning and long-term memory formation (Li and Cai, 2021). It has been reported that miRNA-455-5p/CPEB1 pathway mediated synaptic and memory deficits in Alzheimer’s Disease through targeting on AMPARs (Xiao et al., 2021). Research has shown that the expression of miR-421 is related to neurodegenerative diseases such as Alzheimer’s disease, Parkinson’s disease, and stroke (Yang et al., 2017; Angelopoulou et al., 2019; Ouyang et al., 2022). miR-421 may be involved in the pathogenesis and development of these neurological diseases through mechanisms such as regulating neuron development, metabolism, and immune responses. In neuroscience, TFs are considered important regulatory factors for memory and cognitive function. They can affect the development of neurons, synaptic development, and synaptic plasticity, thereby affecting memory formation and cognitive function. The loss or mutation of transcription factors may be related to neurological disorders and cognitive impairment. The lack of the transcription factor CREB (cAMP response element binding protein) may lead to impaired learning and memory function (Amidfar et al., 2020). The STAT signaling pathway also plays an important role in regulating memory and cognition. STAT3 plays a crucial role in synapse development by controlling axonal guidance and synapse formation, affecting the transmission of neural potentials and memory generation and storage. STAT3 binds to glutamatergic neurotransmitter receptors, regulating glutamate release and function of glutamatergic neurons, thereby affecting memory processing and retrieval (Larsen et al., 2021). Overall, a number of miRNAs as well as TFs were identified that affect expression of these genes, and hence the pathological processes of MCI. The discussion of known effects of targeting these TFs or genetic variations in them in MCI and other neurodegenerative diseases would be helpful and may provide a brand-new research direction for the diagnosis and treatment of MCI and, further, a theoretical basis for the follow-up experimental research on MCI. However, whether MCI patient with over-expression or under-expression of these hub genes could benefit from the regulation of hub genes, or whether these hub genes are promising, therapeutic targets still need further experimental supports including pre-clinical and prospective clinical studies.

There are several limitations in our present study. First, we only used the GEO database data and real samples from a single center for internal as well as external validation, and we still need data from other databases or multiple centers for further validation to test the applicability of the predictive signature. Second, when analyzing the DEGs or CS-DEGs, with the consideration of the complexity of datasets in our study, it is difficult to consider some important factors, such as gender, races, regions, among others. Finally, we found the expression changes of eight hub genes in MCI, nonetheless, the detailed mechanism of such changes was not clear. Therefore, more evidences are required to find out the biological foundation.

Briefly, the cellular senescence-related gene signature can accurately diagnose the occurrence of MCI, they may provide a new avenue for MCI mechanism and treatment.

## Data availability statement

Publicly available datasets were analyzed in this study. This data can be found in the NCBI database, the links are: https://www.ncbi.nlm.nih.gov/geo/query/acc.cgi?acc=GSE63060 and https://www.ncbi.nlm.nih.gov/geo/query/acc.cgi?acc=GSE18309.

## Ethics statement

The studies involving human participants were reviewed and approved by the Medical Ethics Committee of the Third Central Clinical College of Tianjin Medical University (approval number: IRB2022-011-02). The patients/participants provided their written informed consent to participate in this study

## Author contributions

HW designed the study and revised the manuscript. SM analyzed the data, generated the figures and wrote the manuscript. TX analyzed the data, involved in manuscript writing and patients’ recruitment. XW conducted Qrt-PCR and revised the manuscript. All authors contributed to the article and approved the submitted version.

## Funding

This work was supported by grants from the National Natural Science Foundation of China (82071220), Natural Science Foundation of Tianjin (20JCYBJC01290), and the Science and Technology Foundation of Tianjin Health Commission (MS20013), and Tianjin key Medical Discipline (Specialty) Construction Project (TJYXZDXK-072C).

## Conflict of interest

The authors declare that the research was conducted in the absence of any commercial or financial relationships that could be construed as a potential conflict of interest.

## Publisher’s note

All claims expressed in this article are solely those of the authors and do not necessarily represent those of their affiliated organizations, or those of the publisher, the editors and the reviewers. Any product that may be evaluated in this article, or claim that may be made by its manufacturer, is not guaranteed or endorsed by the publisher.

## Footnotes

^1^ https://genomics.senescence.info/cells/

^2^ http://string-db.org/

^3^ https://dgidb.genome.wustl.edu/

^4^ https://www.networkanalyst.ca/

^5^ https://starbase.sysu.edu.cn/index.php

## References

[ref1] Akterin S. Cowburn R. F. Miranda-Vizuete A. Jiménez A. Bogdanovic N. Winblad B.. (2006). Involvement of glutaredoxin-1 and thioredoxin-1 in beta-amyloid toxicity and Alzheimer's disease. Cell Death Differ. 13, 1454–1465. doi: 10.1038/sj.cdd.4401818, PMID: 16311508

[ref2] Alfert A. Moreno N. Kerl K. (2019). The BAF complex in development and disease. Epigenetics Chromatin 12:19. doi: 10.1186/s13072-019-0264-y, PMID: 30898143 PMC6427853

[ref3] Amidfar M. De Oliveira J. Kucharska E. Budni J. Kim Y. K. (2020). The role of CREB and BDNF in neurobiology and treatment of Alzheimer's disease. Life Sci. 257:118020. doi: 10.1016/j.lfs.2020.118020, PMID: 32603820

[ref4] Angelopoulou E. Paudel Y. N. Piperi C. (2019). miR-124 and Parkinson's disease: a biomarker with therapeutic potential. Pharmacol. Res. 150:104515. doi: 10.1016/j.phrs.2019.104515, PMID: 31707035

[ref5] Arodin L. Lamparter H. Karlsson H. Nennesmo I. Björnstedt M. Schröder J.. (2014). Alteration of thioredoxin and glutaredoxin in the progression of Alzheimer's disease. J. Alzheimers Dis. 39, 787–797. doi: 10.3233/JAD-131814, PMID: 24270206

[ref6] Bai L. Zhang S. Zhou X. Li Y. Bai J. (2019). Brain-derived neurotrophic factor induces thioredoxin-1 expression through TrkB/Akt/CREB pathway in SH-SY5Y cells. Biochimie 160, 55–60. doi: 10.1016/j.biochi.2019.02.011, PMID: 30796965

[ref7] Baker D. J. Childs B. G. Durik M. Wijers M. E. Sieben C. J. Zhong J.. (2016). Naturally occurring p16Ink4a-positive cells shorten healthy lifespan. Nature 530, 184–189. doi: 10.1038/nature16932, PMID: 26840489 PMC4845101

[ref8] Balaban R. S. Nemoto S. Finkel T. (2005). Mitochondria, oxidants, and aging. Cells 120, 483–495. doi: 10.1016/j.cell.2005.02.001 15734681

[ref9] Barrera Ocampo Á. A. Céspedes Rubio Á. E. Cardona Gómez G. P. (2008). Mecanismo potencial de neuroprotección y plasticidad sináptica inducidas por el estradiol a través de PI3K/GSK3beta en la isquemia cerebral. Rev. Neurol. 46, 32–39. doi: 10.33588/rn.4601.2007094 18214825

[ref10] Bhaskar K. Yen S. H. Lee G. (2005). Disease-related modifications in tau affect the interaction between Fyn and tau. J. Biol. Chem. 280, 35119–35125. doi: 10.1074/jbc.M505895200, PMID: 16115884

[ref11] Braun S. M. G. Petrova R. Tang J. Krokhotin A. Miller E. L. Tang Y.. (2021). BAF subunit switching regulates chromatin accessibility to control cell cycle exit in the developing mammalian cortex. Genes Dev. 35, 335–353. doi: 10.1101/gad.342345.120, PMID: 33602870 PMC7919417

[ref12] Buccellato F. R. D’Anca M. Fenoglio C. Scarpini E. Galimberti D. (2021). Role of oxidative damage in Alzheimer's disease and neurodegeneration: from pathogenic mechanisms to biomarker discovery. Antioxidants 10:1353. doi: 10.3390/antiox10091353 34572985 PMC8471953

[ref13] Campisi J. Robert L. (2014). Cell senescence: role in aging and age-related diseases. Interdiscip. Top. Gerontol. 39, 45–61. doi: 10.1159/000358899 24862014 PMC4211612

[ref14] Chang J. Hwang H. J. Kim B. Choi Y. G. Park J. Park Y.. (2021). TRIM28 functions as a negative regulator of aggresome formation. Autophagy 17, 4231–4248. doi: 10.1080/15548627.2021.1909835, PMID: 33783327 PMC8726693

[ref15] Chen Z. S. Huang X. Talbot K. Chan H. Y. E. (2021). A fine balance between Prpf19 and Exoc7 in achieving degradation of aggregated protein and suppression of cell death in spinocerebellar ataxia type 3. Cell Death Dis. 12:136. doi: 10.1038/s41419-021-03444-x, PMID: 33542212 PMC7862454

[ref16] Colin-Gonzalez A. L. Paz-Loyola A. L. Serratos I. Seminotti B. Ribeiro C. A. Leipnitz G.. (2015). Toxic synergism between quinolinic acid and organic acids accumulating in glutaric acidemia type I and in disorders of propionate metabolism in rat brain synaptosomes: relevance for metabolic acidemias. Neuroscience 308, 64–74. doi: 10.1016/j.neuroscience.2015.09.002, PMID: 26343296

[ref17] Cortesi M. Zanoni M. Pirini F. Tumedei M. M. Ravaioli S. Rapposelli I. G.. (2021). Pancreatic cancer and cellular senescence: tumor microenvironment under the spotlight. Int. J. Mol. Sci. 23:254. doi: 10.3390/ijms23010254, PMID: 35008679 PMC8745092

[ref18] Cummings J. Lee G. Ritter A. Zhong K. (2018). Alzheimer's disease drug development pipeline: 2018. Alzheimers Dement (N Y) 4, 195–214. doi: 10.1016/j.trci.2018.03.009, PMID: 29955663 PMC6021548

[ref19] Dunne R. A. Aarsland D. O'brien J. T. Ballard C. Banerjee S. Fox N. C.. (2021). Mild cognitive impairment: the Manchester consensus. Age Ageing 50, 72–80. doi: 10.1093/ageing/afaa228, PMID: 33197937 PMC7793599

[ref20] Fatt M. P. Tran L. M. Vetere G. Storer M. A. Simonetta J. V. Miller F. D.. (2022). Restoration of hippocampal neural precursor function by ablation of senescent cells in the aging stem cell niche. Stem Cell Rep. 17, 259–275. doi: 10.1016/j.stemcr.2021.12.010, PMID: 35063124 PMC8828532

[ref21] Fischer A. Sananbenesi F. Wang X. Dobbin M. Tsai L. H. (2007). Recovery of learning and memory is associated with chromatin remodelling. Nature 447, 178–182. doi: 10.1038/nature05772 17468743

[ref22] Friedman J. Hastie T. Tibshirani R. (2010). Regularization paths for generalized linear models via coordinate descent. J. Stat. Softw. 33, 1–22. doi: 10.18637/jss.v033.i01, PMID: 20808728 PMC2929880

[ref23] Gaikwad S. Puangmalai N. Bittar A. Montalbano M. Garcia S. Mcallen S.. (2021). Tau oligomer induced HMGB1 release contributes to cellular senescence and neuropathology linked to Alzheimer's disease and frontotemporal dementia. Cell Rep. 36:109419. doi: 10.1016/j.celrep.2021.109419, PMID: 34289368 PMC8341760

[ref24] Gerszon J. Rodacka A. (2018). Oxidatively modified glyceraldehyde-3-phosphate dehydrogenase in neurodegenerative processes and the role of low molecular weight compounds in counteracting its aggregation and nuclear translocation. Ageing Res. Rev. 48, 21–31. doi: 10.1016/j.arr.2018.09.003, PMID: 30254002

[ref25] Ghosh C. Seal M. Mukherjee S. Ghosh Dey S. (2015). Alzheimer's disease: a Heme-Aβ perspective. Acc. Chem. Res. 48, 2556–2564. doi: 10.1021/acs.accounts.5b00102, PMID: 26252621

[ref26] Glaser A. G. Menz G. Kirsch A. I. Zeller S. Crameri R. Rhyner C. (2008). Auto- and cross-reactivity to thioredoxin allergens in allergic bronchopulmonary aspergillosis. Allergy 63, 1617–1623. doi: 10.1111/j.1398-9995.2008.01777.x, PMID: 19032234

[ref27] Gräff J. Rei D. Guan J. S. Wang W. Y. Seo J. Hennig K. M.. (2012). An epigenetic blockade of cognitive functions in the neurodegenerating brain. Nature 483, 222–226. doi: 10.1038/nature10849, PMID: 22388814 PMC3498952

[ref28] Grinan-Ferre C. Marsal-Garcia L. Bellver-Sanchis A. Kondengaden S. M. Turga R. C. Vazquez S.. (2019). Pharmacological inhibition of G9a/GLP restores cognition and reduces oxidative stress, neuroinflammation and beta-amyloid plaques in an early-onset Alzheimer's disease mouse model. Aging (Albany NY) 11, 11591–11608. doi: 10.18632/aging.102558, PMID: 31804189 PMC6932909

[ref29] Guan J. Haggarty S. Giacometti E. Dannenberg J. Joseph N. Gao J.. (2009). HDAC2 negatively regulates memory formation and synaptic plasticity. Nature 459, 55–60. doi: 10.1038/nature07925, PMID: 19424149 PMC3498958

[ref30] Guerrero A. De Strooper B. Arancibia-Carcamo I. L. (2021). Cellular senescence at the crossroads of inflammation and Alzheimer's disease. Trends Neurosci. 44, 714–727. doi: 10.1016/j.tins.2021.06.007, PMID: 34366147

[ref31] Gupta R. Ambasta R. K. Kumar P. (2020). Pharmacological intervention of histone deacetylase enzymes in the neurodegenerative disorders. Life Sci. 243:117278. doi: 10.1016/j.lfs.2020.117278 31926248

[ref32] Haass C. Mandelkow E. (2010). Fyn-tau-amyloid: a toxic triad. Cells 142, 356–358. doi: 10.1016/j.cell.2010.07.032 20691893

[ref33] Hayflick L. (1965). The limited in vitro lifetime of human diploid cell strains. Exp. Cell Res. 37, 614–636. doi: 10.1016/0014-4827(65)90211-9 14315085

[ref34] Imamura K. Izumi Y. Watanabe A. Tsukita K. Woltjen K. Yamamoto T.. (2017). The Src/c-Abl pathway is a potential therapeutic target in amyotrophic lateral sclerosis. Sci. Transl. Med. 9:eaaf3962. doi: 10.1126/scitranslmed.aaf3962, PMID: 28539470

[ref35] Kadamb R. Mittal S. Bansal N. Batra H. Saluja D. (2013). Sin3: insight into its transcription regulatory functions. Eur. J. Cell Biol. 92, 237–246. doi: 10.1016/j.ejcb.2013.09.001, PMID: 24189169

[ref36] Kanehisa M. Goto S. (2000). KEGG: Kyoto encyclopedia of genes and genomes. Nucleic Acids Res. 28, 27–30. doi: 10.1093/nar/28.1.27, PMID: 10592173 PMC102409

[ref37] Khosla S. Farr J. N. Tchkonia T. Kirkland J. L. (2020). The role of cellular senescence in ageing and endocrine disease. Nat. Rev. Endocrinol. 16, 263–275. doi: 10.1038/s41574-020-0335-y, PMID: 32161396 PMC7227781

[ref38] Kim J. Huang A. Y. Johnson S. L. Lai J. Isacco L. Jeffries A. M.. (2022). Prevalence and mechanisms of somatic deletions in single human neurons during normal aging and in DNA repair disorders. Nat. Commun. 13:5918. doi: 10.1038/s41467-022-33642-w, PMID: 36207339 PMC9546902

[ref39] Kolker S. Sauer S. W. Hoffmann G. F. Muller I. Morath M. A. Okun J. G. (2008). Pathogenesis of CNS involvement in disorders of amino and organic acid metabolism. J. Inherit. Metab. Dis. 31, 194–204. doi: 10.1007/s10545-008-0823-z, PMID: 18392748

[ref40] Langfelder P. Horvath S. (2008). WGCNA: an R package for weighted correlation network analysis. BMC Bioinformatics 9:559. doi: 10.1186/1471-2105-9-559, PMID: 19114008 PMC2631488

[ref41] Larsen S. B. Cowley C. J. Sajjath S. M. Barrows D. Yang Y. Carroll T. S.. (2021). Establishment, maintenance, and recall of inflammatory memory. Cell Stem Cell 28:e1758, 1758–1774.e8. doi: 10.1016/j.stem.2021.07.001 PMC8500942 34320411

[ref42] Lazarev V. F. Tsolaki M. Mikhaylova E. R. Benken K. A. Shevtsov M. A. Nikotina A. D.. (2021). Extracellular GAPDH promotes Alzheimer disease progression by enhancing amyloid-beta aggregation and cytotoxicity. Aging Dis. 12, 1223–1237. doi: 10.14336/AD.2020.1230, PMID: 34341704 PMC8279520

[ref43] Li Q. S. Cai D. (2021). Integrated miRNA-Seq and mRNA-Seq study to identify miRNAs associated with Alzheimer's disease using post-mortem brain tissue samples. Front. Neurosci. 15:620899. doi: 10.3389/fnins.2021.620899, PMID: 33833661 PMC8021900

[ref44] Li Y. Guo X. Sun L. Xiao J. Su S. Du S.. (2020). N(6)-Methyladenosine demethylase FTO contributes to neuropathic pain by stabilizing G9a expression in primary sensory neurons. Adv. Sci. (Weinh) 7:1902402. doi: 10.1002/advs.201902402, PMID: 32670741 PMC7341103

[ref45] Mannervik M. Nibu Y. Zhang H. Levine M. (1999). Transcriptional coregulators in development. Science 284, 606–609. doi: 10.1126/science.284.5414.606 10213677

[ref46] Memoria C. M. Yassuda M. S. Nakano E. Y. Forlenza O. V. (2013). Brief screening for mild cognitive impairment: validation of the Brazilian version of the Montreal cognitive assessment. Int. J. Geriatr. Psychiatry 28, 34–40. doi: 10.1002/gps.3787 22368034

[ref47] Miller M. B. Huang A. Y. Kim J. Zhou Z. Kirkham S. L. Maury E. A.. (2022). Somatic genomic changes in single Alzheimer's disease neurons. Nature 604, 714–722. doi: 10.1038/s41586-022-04640-1, PMID: 35444284 PMC9357465

[ref48] Mitchell A. J. (2009). A meta-analysis of the accuracy of the mini-mental state examination in the detection of dementia and mild cognitive impairment. J. Psychiatr. Res. 43, 411–431. doi: 10.1016/j.jpsychires.2008.04.014, PMID: 18579155

[ref49] Mitchell D. A. Morton S. U. Fernhoff N. B. Marletta M. A. (2007). Thioredoxin is required for S-nitrosation of procaspase-3 and the inhibition of apoptosis in Jurkat cells. Proc. Natl. Acad. Sci. U. S. A. 104, 11609–11614. doi: 10.1073/pnas.0704898104, PMID: 17606900 PMC1913894

[ref50] Mitra A. Vo L. Soukar I. Chaubal A. Greenberg M. L. Pile L. A. (2022). Isoforms of the transcriptional cofactor SIN3 differentially regulate genes necessary for energy metabolism and cell survival. Biochim. Biophys. Acta, Mol. Cell Res. 1869:119322. doi: 10.1016/j.bbamcr.2022.119322, PMID: 35820484 PMC10557476

[ref51] Mladenov E. Li F. Zhang L. Klammer H. Iliakis G. (2018). Intercellular communication of DNA damage and oxidative status underpin bystander effects. Int. J. Radiat. Biol. 94, 719–726. doi: 10.1080/09553002.2018.1434323, PMID: 29377786

[ref52] Muñoz-Espín D. Serrano M. (2014). Cellular senescence: from physiology to pathology. Nat. Rev. Mol. Cell Biol. 15, 482–496. doi: 10.1038/nrm3823 24954210

[ref53] Ogunlade B. Adelakun S. A. Agie J. A. (2022). Nutritional supplementation of gallic acid ameliorates Alzheimer-type hippocampal neurodegeneration and cognitive impairment induced by aluminum chloride exposure in adult Wistar rats. Drug Chem. Toxicol. 45, 651–662. doi: 10.1080/01480545.2020.1754849 32329360

[ref54] Ouyang Q. Liu K. Zhu Q. Deng H. Le Y. Ouyang W.. (2022). Brain-penetration and neuron-targeting DNA Nanoflowers co-delivering miR-124 and Rutin for synergistic therapy of Alzheimer's disease. Small 18:e2107534. doi: 10.1002/smll.202107534 35182016

[ref55] Petr M. A. Tulika T. Carmona-Marin L. M. Scheibye-Knudsen M. (2020). Protecting the aging genome. Trends Cell Biol. 30, 117–132. doi: 10.1016/j.tcb.2019.12.001 31917080

[ref56] Rajan K. B. Weuve J. Barnes L. L. Mcaninch E. A. Wilson R. S. Evans D. A. (2021). Population estimate of people with clinical Alzheimer's disease and mild cognitive impairment in the United States (2020-2060). Alzheimers Dement. 17, 1966–1975. doi: 10.1002/alz.12362, PMID: 34043283 PMC9013315

[ref57] Rousseaux M. W. De Haro M. Lasagna-Reeves C. A. De Maio A. Park J. Jafar-Nejad P.. (2016). TRIM28 regulates the nuclear accumulation and toxicity of both alpha-synuclein and tau. eLife 5:e19809. doi: 10.7554/eLife.19809, PMID: 27779468 PMC5104516

[ref58] Rousseaux M. W. Revelli J. P. Vazquez-Velez G. E. Kim J. Y. Craigen E. Gonzales K.. (2018). Depleting Trim28 in adult mice is well tolerated and reduces levels of alpha-synuclein and tau. eLife 7:e36768. doi: 10.7554/eLife.36768, PMID: 29863470 PMC5993537

[ref59] Saez-Atienzar S. Masliah E. (2020). Cellular senescence and Alzheimer disease: the egg and the chicken scenario. Nat. Rev. Neurosci. 21, 433–444. doi: 10.1038/s41583-020-0325-z, PMID: 32601397 PMC12548380

[ref60] Santen G. W. Aten E. Vulto-van Silfhout A. T. Pottinger C. van Bon B. W. M. van Minderhout I. J. H. M.. (2013). Coffin-Siris syndrome and the BAF complex: genotype-phenotype study in 63 patients. Hum. Mutat. 34, 1519–1528. doi: 10.1002/humu.22394, PMID: 23929686

[ref61] Sikora E. Bielak-Zmijewska A. Dudkowska M. Krzystyniak A. Mosieniak G. Wesierska M.. (2021). Cellular senescence in brain aging. Front. Aging Neurosci. 13:646924. doi: 10.3389/fnagi.2021.646924 33732142 PMC7959760

[ref62] Simone C. (2006). SWI/SNF: the crossroads where extracellular signaling pathways meet chromatin. J. Cell. Physiol. 207, 309–314. doi: 10.1002/jcp.20514, PMID: 16155938

[ref63] Szklarczyk D. Franceschini A. Wyder S. Forslund K. Heller D. Huerta-Cepas J.. (2015). STRING v10: protein-protein interaction networks, integrated over the tree of life. Nucleic Acids Res. 43, D447–D452. doi: 10.1093/nar/gku1003, PMID: 25352553 PMC4383874

[ref64] The Gene Ontology Consortium (2017). Expansion of the Gene Ontology knowledgebase and resources. Nucleic Acids Res. 45, D331–d338. doi: 10.1093/nar/gky1055 27899567 PMC5210579

[ref65] Valko M. Leibfritz D. Moncol J. Cronin M. T. Mazur M. Telser J. (2007). Free radicals and antioxidants in normal physiological functions and human disease. Int. J. Biochem. Cell Biol. 39, 44–84. doi: 10.1016/j.biocel.2006.07.001 16978905

[ref66] Williams A. B. Schumacher B. (2016). p53 in the DNA-damage-repair process. Cold Spring Harb. Perspect. Med. 6:a026070. doi: 10.1101/cshperspect.a026070, PMID: 27048304 PMC4852800

[ref67] Wissler Gerdes E. O. Zhu Y. Weigand B. M. Tripathi U. Burns T. C. Tchkonia T.. (2020). Cellular senescence in aging and age-related diseases: implications for neurodegenerative diseases. Int. Rev. Neurobiol. 155, 203–234. doi: 10.1016/bs.irn.2020.03.019, PMID: 32854855 PMC7656525

[ref68] Xiao G. Chen Q. Zhang X. (2021). MicroRNA-455-5p/CPEB1 pathway mediates Abeta-related learning and memory deficits in a mouse model of Alzheimer's disease. Brain Res. Bull. 177, 282–294. doi: 10.1016/j.brainresbull.2021.10.008, PMID: 34678444

[ref69] Yan M. Zhao Y. Meng Q. Wang S. Ding Y. Liu Q.. (2022). Effects of virtual reality combined cognitive and physical interventions on cognitive function in older adults with mild cognitive impairment: a systematic review and meta-analysis. Ageing Res. Rev. 81:101708. doi: 10.1016/j.arr.2022.101708, PMID: 35953010

[ref70] Yang K. Belrose J. Trepanier C. H. Lei G. Jackson M. F. Macdonald J. F. (2011). Fyn, a potential target for Alzheimer's disease. J. Alzheimers Dis. 27, 243–252. doi: 10.3233/JAD-2011-110353, PMID: 21799250

[ref71] Yang J. Zhang X. Chen X. Wang L. Yang G. (2017). Exosome mediated delivery of miR-124 promotes neurogenesis after ischemia. Mol. Ther. Nucleic Acids 7, 278–287. doi: 10.1016/j.omtn.2017.04.010, PMID: 28624203 PMC5415550

[ref72] Yoon Y. S. You J. S. Kim T. K. Ahn W. J. Kim M. J. Son K. H.. (2022). Senescence and impaired DNA damage responses in alpha-synucleinopathy models. Exp. Mol. Med. 54, 115–128. doi: 10.1038/s12276-022-00727-x, PMID: 35136202 PMC8894476

[ref73] Yu G. Li F. Qin Y. Bo X. Wu Y. Wang S. (2010). GOSemSim: an R package for measuring semantic similarity among GO terms and gene products. Bioinformatics 26, 976–978. doi: 10.1093/bioinformatics/btq064 20179076

[ref74] Zhang X. Pearsall V. M. Carver C. M. Atkinson E. J. Clarkson B. D. S. Grund E. M.. (2022). Rejuvenation of the aged brain immune cell landscape in mice through p16-positive senescent cell clearance. Nat. Commun. 13:5671. doi: 10.1038/s41467-022-33226-8, PMID: 36167854 PMC9515187

